# Using Continuous‐Time Spatial Capture–Recapture models to make inference about animal activity patterns

**DOI:** 10.1002/ece3.6822

**Published:** 2020-10-09

**Authors:** Greg B. Distiller, David L. Borchers, Rebecca J. Foster, Bart J. Harmsen

**Affiliations:** ^1^ Department of Statistical Sciences Centre for Statistics in Ecology, Environment and Conservation (SEEC) University of Cape Town Cape Town South Africa; ^2^ Centre for Research into Ecological and Environmental Modelling School of Mathematics and Statistics University of St Andrews St Andrews UK; ^3^ Panthera New York NY USA

**Keywords:** activity patterns, behavioral ecology, continuous‐time spatial capture–recapture, spatial capture–recapture, temporal partitioning

## Abstract

1. Quantifying the distribution of daily activity is an important component of behavioral ecology. Historically, it has been difficult to obtain data on activity patterns, especially for elusive species. However, the development of affordable camera traps and their widespread usage has led to an explosion of available data from which activity patterns can be estimated.
2. Continuous‐time spatial capture–recapture (CT SCR) models drop the occasion structure seen in traditional spatial and nonspatial capture–recapture (CR) models and use the actual times of capture. In addition to estimating density, CT SCR models estimate expected encounters through time. Cyclic splines can be used to allow flexible shapes for modeling cyclic activity patterns, and the fact that SCR models also incorporate distance means that space–time interactions can be explored. This method is applied to a jaguar dataset.
3. Jaguars in Belize are most active and range furthest in the evening and early morning and when they are located closer to the network of trails. There is some evidence that females have a less variable pattern than males. The comparison between sexes demonstrates how CT SCR can be used to explore hypotheses about animal behavior within a formal modeling framework.
4. SCR models were developed primarily to estimate and model density, but the models can be used to explore processes that interact across space and time, especially when using the CT SCR framework that models the temporal dimension at a finer resolution.

## Graphical abstract

Quantifying the distribution of daily activity is an important component of behavioral ecology, but historically it has been difficult to obtain data on activity patterns. The emergence of camera traps as a mainstream tool in ecological monitoring has led to an explosion of available data from which activity patterns can be estimated. Continuous‐time spatial capture–recapture (CT SCR) models model the encounter process at an arbitrarily fine temporal resolution and so allow users to investigate how activity patterns and space use change continuously over time.

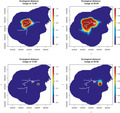

## 1 INTRODUCTION

Animal activity patterns are an important aspect of species ecology that affect community interactions and community structure (Bridges & Noss, 2011; Farris et al., 2015; Frey et al., 2017; Gerber et al., 2012). The way an animal behaves is driven by an energetic trade‐off between different activities. Being active is necessary for sustenance but more costly than resting, and animals need to optimize the way that they expend energy relative to the cost associated with these activities (Frey et al., 2017). For example, predators can maximize their chances of success by hunting when prey are most vulnerable whereas prey may adjust their activity patterns to avoid predation. Adjustments may occur across space when for example wide‐ranging predators have been known to exhibit local adaptations that match the pattern of their prey. Furthermore, adjustments are also known to vary across time for example with differences in night‐time illumination caused by the lunar cycle (Harmsen et al., 2011). Strategies for coexistence usually involve differential preferences for prey, habitat, or distribution of activity levels, but apart from sympatry and predator–prey dynamics, understanding activity patterns provides insight into various other processes such as the effects of local density, anthropomorphic disturbances, seasonal fluctuations of resources, circadian rhythms, and even the degree of synchronicity between peaks in activity (Farris et al., 2015; Frey et al., 2017; Harmsen et al., 2009; Ridout & Linkie, 2009).

Unfortunately, activity is not an easy metric to quantify in natural settings and consequently has received relatively little attention. Historically, data on activity patterns were obtained by directly observing individual animals in the field or in the laboratory. More recently, devices that can track movement (such as radio collars and multiaxial accelerometers) have led to improved ways of measuring activity (Rowcliffe et al., 2014) although usually the cost and effort restricts such studies to a small number of individuals (Harmsen et al., 2009). Timing devices can also be fitted with live traps to provide auxiliary data on the times of capture. For example, Cowan and Forrester (2012) used traps with timing devices to study the behavioral response of possums to capture.

Camera trap surveys are a noninvasive way to “capture” or detect individuals and can be applied in a wide range of settings to generate data on multiple species, including species that are difficult to capture or observe directly. Compared with traditional approaches, it is now easier to collect data on a range of species including carnivores, ungulates, and rodents. Camera traps have been used in the field as remote sensors since the early 20th century but technological advancements over the past two decades have made these devices less expensive and more effective, becoming a mainstream tool in ecological research. Data obtained from camera traps are being used for various types of analyses including studies of species richness, occupancy models, and capture–recapture (CR) models (both spatial and nonspatial) (Harmsen et al., 2009; Ridout & Linkie, 2009; Rowcliffe & Carbone, 2008; Rowcliffe et al., 2008; Steenweg et al., 2017).

Data from camera traps are generated in continuous‐time. They usually consist of photographs along with a date and time stamp and therefore contain information that can be used to make inference about activity pattern. Using data from camera trap surveys, Harmsen et al. (2009) examine histograms of hourly capture times to make inference about the hourly activity of jaguars and pumas (as well as their major prey species (Harmsen et al., 2011)). Others have used kernel density estimation for circular data, or fitted circular distributions (such as the Von Mises distribution) to the data, to produce estimators of the continuous activity pattern distributions (Farris et al., 2015; Linkie & Ridout, 2011; Oliveira‐Santos et al., 2013; Ridout & Linkie, 2009; Rowcliffe et al., 2014). Current approaches for quantifying activity patterns from temporal data have made progress but exploring how different covariates affect activity remains a challenge (Frey et al., 2017) and the lack of a standardized methodology has limited research into activity patterns.

In the past, researchers conducting surveys of wildlife populations would sample the population of interest at discrete points in time (for example at the start of the breeding season). Such a study design leads to clear and well‐defined occasions and is the primary reason why traditional CR and spatial capture–recapture (SCR) models have an occasion structure. However, in contrast to these traditional surveys, devices like camera traps sample continuously in time. The growing usage and importance of camera trap surveys have led to the development of a continuous‐time (CT) SCR model that operates with times of detection (Borchers et al., 2014; Distiller, 2016; Distiller & Borchers, 2015).

While the primary aim of SCR models was to estimate and model animal density, it is possible to use these models to learn about other processes and the CT SCR framework provides a formal modeling framework to explore different hypotheses about activity patterns. Furthermore, understanding the complexities of interactions between space and time use for individuals within populations is important to understanding variation between populations in terms of general ecology and distribution, as we would expect that differences in the timing of activity will interact with space use. Understanding this interaction is essential for understanding the spatial distribution of species ecology in detail.

This paper uses data from a camera trap survey of jaguars in Belize to demonstrate how CT SCR models can be used to make inference about animal activity patterns. Initially, models are fitted to detections of male jaguars only and then subsequently to data on both sexes to illustrate how sex‐specific encounter rate functions can be estimated. Subsection 2.1 provides further details of the data, and Subsection 2.2 introduces the CT SCR model with different ways to parameterize the encounter rate function. The results are shown in Section 3, and the discussion appears in Section 4.

## 2 MATERIALS AND METHODS

### 2.1 Study site and camera trapping of jaguars

**FIGURE 1:**
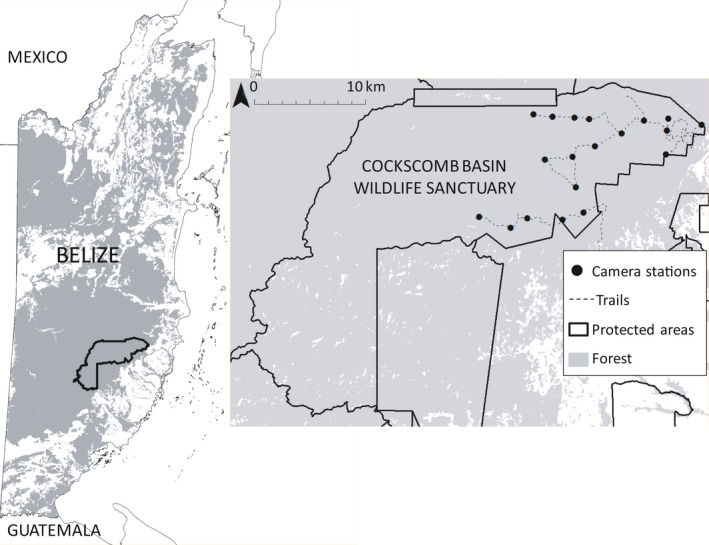
Camera trap survey sites within Cockscomb Basin Wildlife Sanctuary, Belize.

The Cockscomb Basin Wildlife Sanctuary in Belize encompasses 490 km^2^ of secondary tropical moist broadleaf forest at various stages of regeneration following anthropogenic and natural disturbance (for more details see Harmsen et al., 2010b). To the west, the sanctuary forms a contiguous forest block with the protected forests of the Maya Mountain Massif (≈5,000 km^2^ of forest). To the east, the sanctuary is partially buffered by unprotected forest beyond which is a mosaic of pine savannah, shrub land, and broadleaf forest, interdispersed with villages and farms. Jaguars (*Panthera onca*) are found throughout this landscape (Foster et al., 2010). There are 65 km of trails, all within the eastern part of the sanctuary (Figure 1).

Twenty paired camera stations (Pantheracam v3) were deployed along the trail network within the eastern basin. Neighboring stations had an average spacing of 2.0 km (1.1–3.1 km), and digital photographic data were downloaded every 2 weeks. The data used in this paper include 287 detections of 19 individual male jaguars, and 44 detections of eight individual female jaguars, over a 6‐month period (August 2013 to February 2014).

### 2.2 Continuous‐Time Spatial Capture–Recapture (CT SCR)

Suppose that data are generated from a survey of duration *T* that uses an array of *J* camera traps. The primary difference from the discrete‐time (DT) model is that there are no longer *K* occasions. For the *i*th detected individual, instead of a capture history of length *K* there are *ω_ij_* captures at detector *j* at times *t_ij_*_ _= (*t_ij_*_1_, …, *t*_ij_ *_ωij_*). In addition, there are *n* unique animals where the two‐dimensional coordinates of the *i*th individual's activity center are notated as ***s*** *_i_*, and the distance from an individual's activity center to the *j*th detector (*d_j_* (***s*** *_i_*)) affects detectability. Note that the dependence of detection on distance through ***s*** is not always made explicit in what follows.

The observation process in the DT SCR formulation can be parameterized to use either a detection function or an encounter rate function. For an occasion of some specified length, and for an individual whose activity center is a certain distance away from the camera trap, the detection function estimates the probability of detecting the individual whereas the encounter rate function estimates the expected number of encounters (Efford et al., 2013).

CT models generalize the encounter rate function so that, in addition to space, detectability also depends on time. Consequently, the expected encounter rate for the *i*th individual and the *j*th detector at time *t* now depends on both space and time and is denoted as *λ_j_* (*t*, ***s*** *_i_*; ***θ***), where ***θ*** is an unknown vector of encounter rate function parameters. Note that *λ*_0_ is used to denote the intercept of the encounter rate function (see Section 2.2.1 below) and not the expected encounter rate for the *j*th detector where *j* = 0.

The CT framework models the process generating detections as a nonhomogeneous temporal Poisson process where the events of interest are detections. The “survivor function” for individual *i* at detector *j* over the whole survey (the probability of individual *i* not being detected by detector *j* by time *T*) is

(1) $$S_{j}\;\left(T,\;s_{i};\;\theta \right)\;=\;\exp\;\left(‐\int_{0}^{T}\lambda_{j}\;(t,\;s_{i};\;\theta \right)\;dt)$$

and hence $1‐S_{j}\;\left(T,\;s_{i};\;\theta \right)$ is the probability of detection during the period (0, *T*). Similarly, the combined detection hazard over all *J* detectors at time *t* is $\lambda .\left(t,\;s_{i};\;\theta \right)\;=\;\sum_{j=1}^{J}\lambda_{j}\;(t,\;s_{i};\;\theta )$, and the overall probability of detection in (0, *T*) over all detectors is $p.\left(s_{i};\;\theta \right)\;=\;1‐S.\left(T,\;s_{i};\;\theta \right)$, where $S.\left(T,\;s_{i};\;\theta \right)\;=\;\exp\;\left(‐\int_{0}^{T}\lambda .(t,\;s_{i};\;\theta \right)\;dt)$ is the overall survivor function.

Assuming that, conditional on the activity center location, the times of detection are independent, the detection times for individual *i* at detector *j* can be modeled with pdf:

(2) $$f\;\left(t_{\mathrm{ij}}|s_{i};\;\theta \right)\;=\;S_{j}\;\left(T,\;s_{i};\;\theta \right)\;\prod_{r\;=\;1}^{\omega_{\mathrm{ij}}}\lambda_{j}\;(t_{\mathrm{ijr}},\;s_{i};\;\theta )$$

Furthermore, we assume that the number of individuals detected during the survey is a Poisson random variable with intensity $\Lambda \left(\theta ,\phi \right)=\int_{A}D\left(x;\phi \right)p_{\cdot }\left(x;\theta \right)dx$ where *D* () is a function for animal density, *x* is the two‐dimensional coordinate of any location in the study area, and *ϕ* is a vector of unknown parameters that govern the intensity and hence the distribution of activity centers.

The CT SCR likelihood can then be shown (Borchers et al., 2014; Distiller, 2016) to be:

(3) $$L\left(\phi ,\theta |n,t\right)=\frac{e^{‐\Lambda (\phi ,\theta )}}{n!}\prod_{i=1}^{n}\int_{A}D\left(s_{i};\phi \right)\prod_{j=1}^{J}S_{j}\left(T,s_{i};\theta \right)\prod_{r=1}^{\omega_{\mathrm{ij}}}\lambda_{j}\left(t_{\mathrm{ijr}},s_{i};\theta \right)ds$$

#### 2.2.1 The CT encounter rate function

Detectability now depends on both space and time. Suitable functional forms for *λ* as a function of time will depend on the problem at hand. In general, the encounter rate is not expected to increase or decrease monotonically with time and regression splines may be useful to model flexible forms of dependence (Wood, 2017). The focus here is on cyclical functions that repeat themselves after a specified cycle duration and are constrained to transition smoothly between cycles. With a cycle duration of 24 hr, we can model daily patterns in detectability that arise from daily cycles in animal behavior. For example, nocturnal animals are more likely to be detected during the night than the day, and to transition smoothly between being more and less detectable, so that a smooth function of time is appropriate.

There are various ways that one can make the encounter rate function depend on time. The most commonly used form is that of a half‐normal which is defined as *λ*_0_ exp (‐*d* ^2^/2σ^2^)) where *λ*_0_ is the intercept parameter and σ the scale parameter that determines how quickly detectability decreases with distance. It is common practice to make one or both of these parameters depend on covariates, and as time is just another covariate, we can parameterize dependence on time in this way too. Note that the *ij* notation is suppressed in what follows for brevity.

##### **Time‐dependent** *λ*_0_

The standard DT expected encounter rate function has this form:

(4) $$\lambda \;\left(d\right)\;=\;\lambda_{0}\tilde{\lambda }\;\left(d\right)$$

where $\tilde{\lambda }\;\left(0\right)\;=\;1$. Here, *λ*_0_ is the expected number of encounters per unit time of an animal with activity center at the detector and the function $\tilde{\lambda }\;\left(d\right)$ is sometimes called a “kernel”; it might have half‐normal form, for example. We can model the dependence of *λ*_0_ on time, using regression splines, as follows:

(5) $$\lambda_{0}\;\left(t\right)\;=\;\exp\;\left\{\beta_{0}\;+\;\sum_{v\;=\;1}^{V}\beta_{v}\;b_{v}\;\left(t\right)\right\}$$

where *b_v_* (*t*) (*v* = 1, … *V*) is a set of (possibly cyclic) basis functions. If exp {*β*_0_} is written as *λ*_0_ the encounter rate function can be written as

(6) $$\lambda \left(d,t\right)=\lambda_{0}\left(t\right)\tilde{\lambda }\left(d\right)=\lambda_{0}e^{\left(\sum_{v=1}^{V}\beta_{v}b_{v}\left(t\right)\right)}\tilde{\lambda }\left(d\right)=\left(\lambda_{0}\tilde{\lambda }\left(d\right)\right)e^{\left(\sum_{v=1}^{V}\beta_{v}b_{v}\left(t\right)\right)}$$

where $\tilde{\lambda }\;\left(d\right)$ is an encounter rate kernel that depends on distance.

In this case, distance just scales the encounter rate function but does not change its shape across time, and $\exp\;\left\{\sum_{v\;=\;1}^{V}\beta_{v}\;b_{v}\;\left(t\right)\right\}$ determines the shape of the encounter rate function over time. With this parameterization, the *β*_0_ spline intercept is redundant as it gets absorbed into the $\tilde{\lambda }\;\left(d\right)$ term, and a degree of freedom is lost when specifying functions with a cyclic cubic spline form.

When the encounter rate function is parameterized as above, it can be shown that there is no information in the detection times about density (Distiller, 2016). Therefore, if the underlying data are being generated from such a process, a DT model estimator that ignores the capture times will not be biased for density (Distiller, 2016). However, detection times do still contain information about how activity patterns change with time (if detection is by virtue of movement, for example), and this may be of interest in itself.

##### Time‐dependent *σ*

Models with *σ* being time‐dependent have animals propagating themselves over smaller distances at some times than at others. For example, an encounter rate function with a half‐normal shape and a cyclical time effect on the range parameter, *σ* would be specified as follows:

(7) $$\lambda \;\left(d,\;t\right)\;=\;\lambda_{0}\;\exp\;\left\{‐d^{2}/\left(2\sigma \;{\left(t\right)}^{2}\right)\right\}$$

where $\sigma \;\left(t\right)\;=\;\exp\;\left(\beta_{0}\;+\;\sum_{v\;=\;1}^{V}\beta_{v}\;\times \;b_{v}\;\left(t\right)\right)$

Specifying an encounter rate model where the range of the encounter rate function changes with time leads to a function in which the effects of time and distance interact. Consequently, one needs to assess the effect of time (or distance) for a given value of distance (or time), or else to view the encounter rate function in three dimensions.

Equation (7) uses a *λ*_0_ intercept that is constant through time. It is possible to extend the model to allow both *λ*_0_ and *σ* to depend on time. Another option is to use parameterizations similar to those of Efford and Mowat (2014) to build in a complementary relationship between *λ*_0_ and *σ* *a*s functions of time.

#### 2.2.2 Ecological distance

Standard SCR models use ordinary straight‐line Euclidean distance but have been integrated with resource selection information and with an ecological distance metric in order to learn about space usage and landscape connectivity (Fuller et al., 2016; Royle, Chandler, Gazenski, & Graves, 2013; Royle, Chandler, Sun, & Fulle, 2013; Sutherland et al., 2015). Ecological distance can be calculated using least‐cost path algorithms that depend on a conductance parameter that is associated with a salient landscape feature. For example, jaguars routinely walk the trail system as doing so is easier than moving through the dense vegetation (Harmsen et al., 2010a) and hence the distance to the trail network may be a relevant covariate.

Incorporating ecological distance means that models can take account of the habitat in a survey area and recognize that the least‐cost distance between a detector and activity center depends on the habitat in their vicinity and that this distance may be different from the Euclidean distance. Using ecological distance also provides the model with the flexibility to move away from circular home ranges. Lastly, the conductance parameter can be included in the likelihood and estimated in the same way as the other parameters.

#### 2.2.3 Fitted models

The utility of the method presented here is demonstrated by fitting several different types of models (Table 1). Firstly, models with different parametrizations and different distance metrics are fitted to the data on male jaguars only. The objective of this set of models is to illustrate the different types of encounter rate functions and inferences that can be made.

**TABLE 1:** Models fitted in this analysis

Model	Data	Distance metric	*df*s
*λ*_0_ (*t*)	Males only	Euclidean	2, 4, 6
*σ* (*t*)	Males only	Euclidean	2, 4, 6
*σ* (*t*)	Males only	Ecological	4
*λ*_0_ (*t*)	Both sexes	Euclidean	2 (F), 4 (M, Common)

Note

The various combinations of model parameterization, data, and distance metric are shown along with the number of spline parameters (*df*s) from the encounter rate function that are estimated.

Secondly, models are fitted to data on both sexes. In addition to the detections of male jaguars, the survey also produced 44 detections of eight female jaguars and hence these data can be used to illustrate the estimation and comparison of sex‐specific activity patterns. CT SCR models can be used to explore differences in activity patterns between different sexes, as well as different species, time periods, or locations.

#### 2.2.4 Model fitting

All models are fitted using a maximum likelihood framework using the nlm optimizer in R (R Core Team, 2019), and the Aikaike Information Criteria corrected for small sample size (AICc) (Burnham & Anderson, 2002) are used to assess the degree of support in the data for different hypothesized model structures. The time‐dependent component of the encounter rate function is modeled here with a cyclic cubic spline with a cycle duration of 24 hr. We used the R package *mgcv* (Wood, 2014) to construct the necessary basis functions. The degrees of freedom (*df*) that are reported here match the actual number of spline parameters that are estimated. The integral in Equation 3 is evaluated numerically.

Standard errors for individual parameters are obtained from inverting the Hessian matrix. The expected encounter rate depends on several parameters, and the Delta method is used to approximate the variance on the log scale. The confidence limits on the encounter rate scale are produced by appropriately transforming the limits on the log scale. Lastly, computations are performed using facilities provided by the University of Cape Town's ICTS High Performance Computing team (http://hpc.uct.ac.za).

## 3 RESULTS

The results are presented firstly from fitting models to data on male jaguars only. Both parameterizations from subsection 2.2.1 with conventional Euclidean distance are included, and Equation 7 is also used with ecological distance. Thereafter, results from an analysis using the *λ*_0_ (*t*) form on data from both sexes follow.

### 3.1 Male jaguars only

#### **Models with** *λ*_0_ (*t*)

**FIGURE 2:**
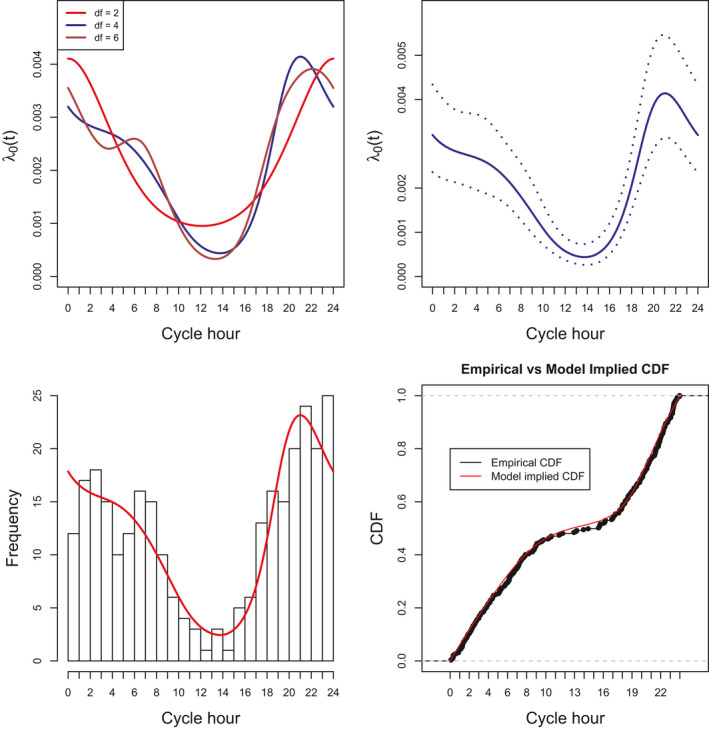
Estimated encounter rate functions using the *λ*_0_ (*t*) parameterization with a 24‐hr cycle fitted to the male jaguar data. Top left panel: *λ*_0_ (*t*) components illustrate how the estimated shape through time changes as *df* (level of complexity) increases, *df* = 2, 4, and 6; top right panel: the 95% confidence interval for the function with *df* = 4. Bottom left panel: the observed detection times collapsed on to one cycle with the model estimated encounter rate function overlaid (red) and scaled to have the same area under the curve as the histogram. Bottom right panel: the empirical and the model implied Cumulative Distribution Functions.

The best‐fitting model of encounter rate used a function with *df* = 4 (Table 2, Figure 2) suggesting that an encounter rate function with an evening peak that falls away slowly during the early morning is most appropriate for modeling the detection of male jaguars in the Cockscomb Basin Wildlife Sanctuary in Belize.

**TABLE 2:** Density estimates and ΔAICc’s from models with time‐varying *λ*_0_. 95% confidence intervals are included

	Density (per 100 km^2^)	95% CI	ΔAICc
*df* = 4	2.17	1.35–3.49	0.00
*df* = 6	2.17	1.35–3.49	10.09
*df* = 2	2.17	1.35–3.49	14.92

The top panels in Figure 2 depict the *λ*_0_ (*t*) component of the encounter rate function for a 24‐hr cycle, that is, there is no effect of distance and the focus is on the shape of the encounter rate function through time. Expected encounter rates can also be plotted for a given distance and for any duration of time. The estimated encounter rate function provides a good fit to the temporal pattern of detections observed in the data (Figure 2, bottom panels).

#### Models with *σ* (*t*)

We also fit models that use the specification given in Equation (7) for three levels of complexity. The density estimates from these models are now slightly different from each other, and the model with *df* = 4 is again selected as the best on the basis of AICc (Table 3).

**TABLE 3:** Density estimates and ΔAICc's from models with time‐varying *σ*. 95% confidence intervals are included

	Density (per 100 km^2^)	95% CI	ΔAICc
*df* = 4	2.13	1.32–3.43	0.00
*df* = 6	2.18	1.36–3.50	9.28
*df* = 2	2.08	1.29–3.37	13.96

**FIGURE 3:**
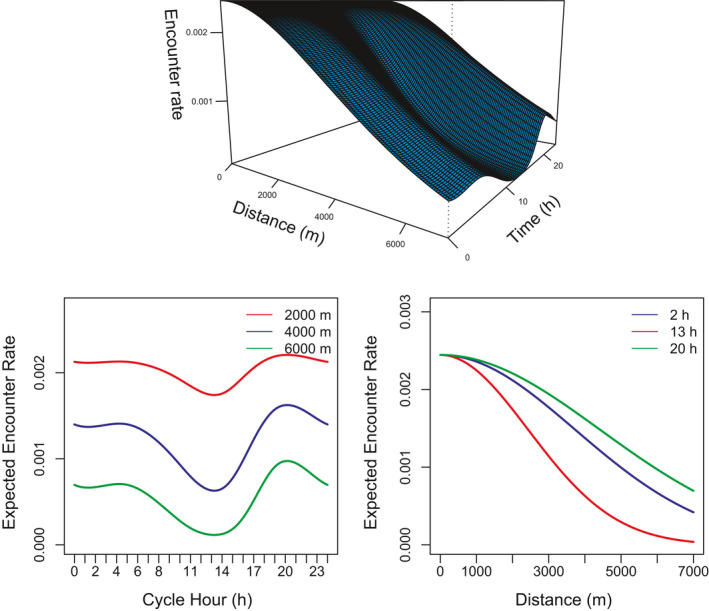
The time and distance dependent expected encounter rate function fitted to the male jaguar data. Top panel: the function in 3 dimensions. Bottom panels: the effect of time (distance) for a given value of distance (time).

Visualizing the estimated encounter rate function is more complicated than for the *λ*_0_ (*t*) parameterization because of the interactive effect of time and distance on the expected encounter rate. The fitted encounter rate function can be viewed as a function of both time and distance in a 3‐dimensional plot (top panel: Figure 3), or 2‐dimensional plots can be used to assess the effect of time (or distance) for a specified value of distance (or time) (bottom panels: Figure 3).

The estimated encounter rate has a peak at around 20:00 though the differences between the peaks and troughs increase as distance from the detector increases. Similarly, it is apparent that at times of the day when an animal does not range far (and so has a low value for *σ*) the expected encounter rate falls away much more quickly with distance than at times that correspond to higher values for *σ*. The AICc's suggest that the simpler parameterization is preferred (Table 4).

**TABLE 4:** Comparing models with *λ*_0_ (*t*) and *σ* (*t*). 95% confidence intervals are included

	Density (per 100 km^2^)	95% CI	ΔAICc
*λ*_0_ (*t*)	2.17	1.35–3.49	0.00
*σ* (*t*)	2.13	1.32–3.43	33.30

#### Using ecological distance

A model with the *σ* (*t*) parameterization that includes ecological distance based on a distance to the trail system covariate produces an estimate for conductance of −1.7, that is, locations further from the trail network are less suitable to move through.

**FIGURE 4:**
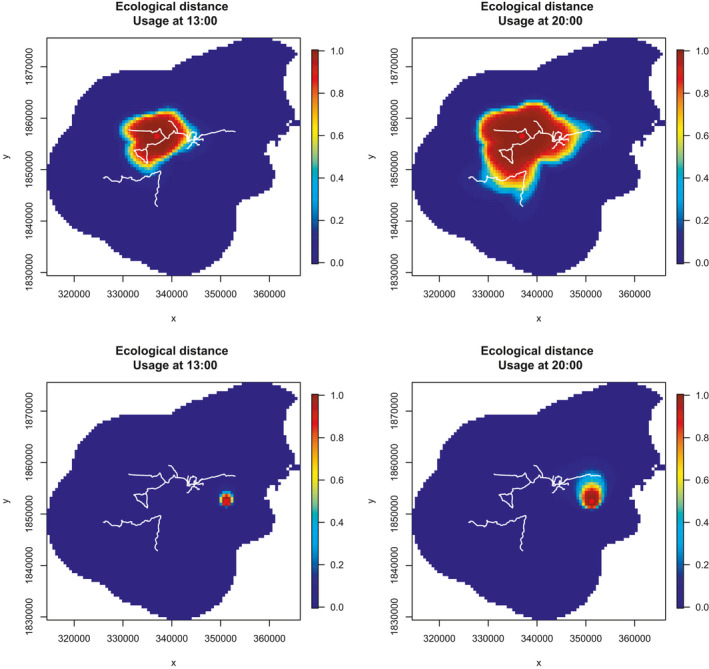
Estimated relative space usage at two different times (13:00 and 20:00) for two animals with activity centers at two different places marked by red dots. The trail system is shown in white.

If space is discretized into pixels, the probability of detecting an individual in a pixel, were there a detector in the pixel, can be used as a proxy for the individual's use of the pixel. Figure 4 shows the estimated relative space usage at two times of day for two animals with activity centers at two different places. Comparing the left and right plots, one can see how the animal is expected to roam further when it is estimated to be more active. Also an animal with an activity center close to the network of trails is estimated to roam further than one with a center that is away from the trails and that the shape of the usage area can be irregular.

### 3.2 Sex‐specific comparison

The *λ*_0_ (*t*) parameterization given in Equation (6), with a cycle duration of 24 hr, is used to fit two models. The first model specifies a common encounter rate function for both sexes whereas the 2nd model estimates a different encounter rate function for each sex. A spline with *df* = 4 is used for models that specify a common encounter rate function whereas the sex‐specific functions use the same value for the male data (*df* = 4) and *df* = 2 for the female data. Note that models with a variety of different dfs were fitted to the female data and clearly showed that *df* = 2 is appropriate. The models also specify a different *σ* value for males and females since it is well known that male jaguars tend to range further than females (the estimates here are *σ*_m_ = 3,721 m and *σ*_f_ = 1,732 m).

**FIGURE 5:**
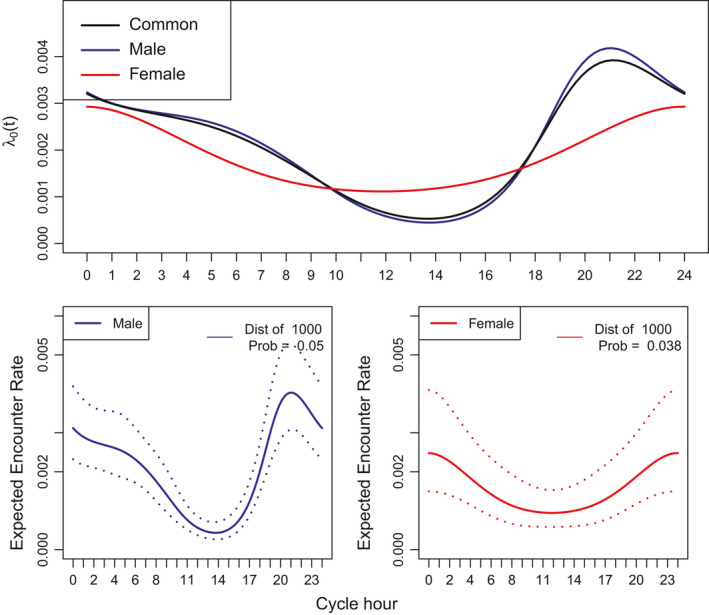
Top panel: estimated *λ*_0_ (*t*) component from the encounter rate function for both sexes together (common, black line) and from the model with sex‐specific functions (male, blue line; female, red line), separate values for *σ* are specified in both cases. Bottom panels: estimated sex‐specific encounter rate functions for a distance of 1 km with 95% confidence intervals.

The expected encounter rate for females is less variable than for males, and females are more likely than males to be detected during the middle part of the day and less likely than males to be detected at night. Furthermore, female activity at night peaks later than the male activity (midnight rather vs. 21:00) (Figure 5). Based on statistical criteria, the two encounter rate functions are not sufficiently different to justify estimating extra parameters; for these data, a common encounter rate function is preferred (Table 5). Of course, there may be biological interest in estimating separate patterns despite the AICc results.

**TABLE 5:** ΔAICc's from models with *λ*_0_ (*t*)

	Common hazard	Separate hazard
AICc	0.00	4.48

## 4 DISCUSSION

The emergence and rapid growth in usage of camera traps has greatly increased the generation of data that can provide information on activity patterns. CT SCR models can be used to estimate encounter rates through time from data that contain capture times, and to make inference about activity levels in relation to space use. The CT SCR framework utilizes the actual times of detection and provides a flexible model‐based method of estimating activity patterns and a rigorous framework for model selection and uncertainty quantification. The ability to use standard model selection tools within a formal modeling framework allows researchers to evaluate the extent of support for competing hypotheses related to animal activity across space or time.

The simplest way to estimate an activity pattern from data on detection times is to summarize the information with histograms as done by Harmsen et al. (2009), for example. Alternatively, it is possible to use a simpler modeling approach (like fitting a regression spline to the histogram data). This discards the information on which individuals were detected where and when. By contrast, the CT SCR formulation provides a modeling framework that uses data at the individual level, is able to accommodate models for changes in activity patterns with both spatial and nonspatial explanatory variables, and provides a framework for testing hypotheses about the drivers of activity patterns. We have shown how this can be done using “ecological distance” and modeling the conductance of the environment. However, a limitation of the example here is that if a pixel with a large distance from the trails is selected, the model estimates that the usage will hardly extend beyond that chosen point, for example, the model estimates what may be an unrealistically low usage for points far away from the trail network. It is likely that this is related to the study design because all cameras are on the trails and so there is no sampling at distances away from the trail network. The issue of what constitutes a good design for drawing inferences about spatio–temporal activity patterns remains to be explored.

The utility of camera traps for detecting a wide array of species means that multi‐species analyses of space and time use are also possible, enhancing the study of predator–prey interactions and interspecific competition. For example, separate analyses of the use of space and time in studies of interspecific avoidance, like those found for jaguars and pumas (Harmsen et al., 2009), might be more robust using a spatio–temporal framework like that presented here. Furthermore, the use of time and space by carnivore species may vary with a range of social and demographic factors, some of which may be visually distinguishable from camera trap photographs (e.g., sex, body size, external body condition). Others may be indistinguishable (e.g., reproductive status, health) or only assigned following long‐term camera monitoring (e.g., social status, age). Our framework has the potential to identify individual variation in the use of space through time and hence distinguish individual‐level characteristics without the need for long‐term monitoring.

Ridout and Linkie (2009) present 12 hypothetical activity patterns that represent the sorts of patterns one is likely to see in realistic biological settings, and Farris et al. (2015) present estimated activity patterns for nine different species that have very different shapes. The key characteristics of these patterns are diurnal versus nocturnal, the number of peaks, the intensity of the peak(s), and the extent to which activity levels reduce during inactive periods. Extensive simulations that explore encounter rate functions of different complexity, in conjunction with different density models, confirm that CT models are able to estimate the underlying encounter rate functions well (Borchers et al., 2014; Distiller, 2016). Hence, CT SCR models can accurately estimate a variety of encounter rate function shapes and with adequate sample size should be able to detect features of interest and to investigate various questions of biological interest related to activity. For example, data collected on the same species at different times of the year or at different locations could be used to investigate seasonal differences in activity patterns or how local factors affect behavior, whereas data on two different species at the same time of year could be used to explore sympatric partitioning of time.

The encounter rate functions presented here all used a repeating cycle of 24 hr. It is possible that detectability can also depend on cycles with a longer duration. For example, a fuller moon leads to brighter hunting conditions and Harmsen et al. (2011) report that the two main prey species of jaguars and pumas both exhibited reduced activity under these conditions and discuss different potential predator strategies in response to this reduction in activity. Note however that it is likely that if such a process existed it would be on top of a shorter daily detection cycle and both would need to be modeled together.

There is evidence that jaguars living in a protected forest in Belize are most active in the evening and early morning. There is also some evidence that females have a slightly different pattern to males with fewer peaks and troughs and with their activity peaking later than males. There are various hypotheses that could explain such temporal avoidance: males need to maximize their access to females and consequently traverse greater distances than females and so tend to use the trail system when it is coolest; when females are not in estrus they avoid males and may use the trail system less extensively during peak male travel times; while females tend to hunt smaller prey, they still have to provide food for dependents and in order to fulfill their energetic needs may potentially need to be active for longer periods than males. It is also apparent that there is virtually no difference in the density estimates from the λ_0_ (*t*) models with differing levels of complexity. Recall that a consequence of the specification of the encounter rate function used here is that the capture times do not have any information about density. It is therefore not surprising that the different model specifications for the observation process do not lead to different estimates of density. In contrast to this, the σ (*t*) models produce slightly different estimates for density although model selection statistics suggest that the extra complexity of the 3‐dimensional encounter rate function is not necessary (Table 4). Based on patterns from monthly capture rates across the camera trap array, Harmsen et al. (2009) found evidence for temporal avoidance between jaguars and pumas and found that both species used the same areas and temporal activity patterns but avoided the same location (detector) at the same time. In the applications presented here, a single activity schedule is estimated for each sex but it is straightforward to extend the model to estimate species‐specific encounter rate functions for different months if required.

Spatial capture–recapture models were developed to estimate density; however, these models open up opportunities to learn about other spatio–temporal processes (Borchers & Fewster, 2016). If the aim is to make inference not only about density but also about space usage, movement or some other spatio–temporal process, then the estimation of the observation process is of direct interest in its own right, and the ability of the CT models to flexibly model the observation process adds a new dimension to SCR models. CT SCR models have the potential to provide a valuable tool for researchers trying to understand animal activity patterns and space usage.

## CONFLICT OF INTEREST

None declared.

## AUTHOR CONTRIBUTIONS

**Greg Distiller:** Conceptualization (equal); Formal analysis (lead); Methodology (equal); Writing–original draft (lead); Writing–review and editing (lead). **David Borchers:** Conceptualization (equal); Methodology (equal); Writing–original draft (supporting); Writing–review and editing (supporting). **Bart J Harmsen:** Data curation (lead); Writing–original draft (supporting); Writing–review and editing (supporting). **Rebecca Foster:** Data curation (lead); Writing–original draft (supporting); Writing–review and editing (supporting).

## Data Availability

The camera trap survey data will be archived on Dryad (https://doi.org/10.5061/dryad.n2z34tmv2).
